# Disintegrins from Hematophagous Sources

**DOI:** 10.3390/toxins4050296

**Published:** 2012-04-26

**Authors:** Teresa C. F. Assumpcao, José M. C. Ribeiro, Ivo M. B. Francischetti

**Affiliations:** Vector Biology Section, Laboratory of Malaria Vector Research, National Institute of Allergy and Infectious Diseases, National Institutes of Health, Bethesda, MD 20852, USA

**Keywords:** disintegrins, bloodsucking, sialome, sialogenins, platelet aggregation, angiogenesis, snake venom, hematophagy, thrombus, transcriptome, proteome, salivary

## Abstract

Bloodsucking arthropods are a rich source of salivary molecules (sialogenins) which inhibit platelet aggregation, neutrophil function and angiogenesis. Here we review the literature on salivary disintegrins and their targets. Disintegrins were first discovered in snake venoms, and were instrumental in our understanding of integrin function and also for the development of anti-thrombotic drugs. In hematophagous animals, most disintegrins described so far have been discovered in the salivary gland of ticks and leeches. A limited number have also been found in hookworms and horseflies, and none identified in mosquitoes or sand flies. The vast majority of salivary disintegrins reported display a RGD motif and were described as platelet aggregation inhibitors, and few others as negative modulator of neutrophil or endothelial cell functions. This notably low number of reported disintegrins is certainly an underestimation of the actual complexity of this family of proteins in hematophagous secretions. Therefore an algorithm was created in order to identify the tripeptide motifs RGD, KGD, VGD, MLD, KTS, RTS, WGD, or RED (flanked by cysteines) in sialogenins deposited in GenBank database. The search included sequences from various blood-sucking animals such as ticks (e.g., *Ixodes* sp., *Argas* sp., *Rhipicephalus* sp., *Amblyomma*sp.), tabanids (e.g., *Tabanus* sp.), bugs (e.g., *Triatoma* sp., *Rhodnius prolixus*), mosquitoes (e.g., *Anopheles* sp., *Aedes* sp., *Culex* sp.), sand flies (e.g., *Lutzomyia* sp., *Phlebotomus* sp.), leeches (e.g., *Macrobdella* sp., *Placobdella* sp.) and worms (e.g., *Ancylostoma* sp.). This approach allowed the identification of a remarkably high number of novel putative sialogenins with tripeptide motifs typical of disintegrins (>450 sequences) whose biological activity remains to be verified. This database is accessible online as a hyperlinked worksheet and displays biochemical, taxonomic, and gene ontology aspects for each putative disintegrin. It is also freely available for download (right click with the mouse) at links http://exon.niaid.nih.gov/transcriptome/RGD/RGD-Peps-WEB.xlsx (web version) and http://exon.niaid.nih.gov/transcriptome/RGD/RGD-sialogenins.zip (stand alone version).

## 1. Introduction

Integrins are a large family of heterodimeric adhesion receptors that are formed by non-covalent association of different α and β chains and mediate cell adhesion to ECM (extracellular matrix) proteins as well as cell-cell interactions. Many integrins bind to their extracellular ligands through the recognition of the tripeptide Arg-Gly-Asp (RGD) flanked by disulphide bonds that form a peptide hairpin with the G at its apex, flanked by amino acids of opposite charges. This motif is present in several protein components of extracellular matrix (e.g., vitronectin, fibronectin, osteopontin, and fibrinogen). Although different ligands can bind to the same integrin, sequences flanking the RGD peptide are reported to be important for integrin specificity. Integrin ligation by its natural ligands promotes intracellular signaling by activating a number of intracellular mediators that ultimately lead to cell migration, survival, and invasion [1,2,3]. Remarkably, the mechanism of action of integrins has been much advanced by the discovery of peptides from viper venoms, known as the disintegrins, which reportedly block integrin interaction with its physiological ligands present in the plasma or in the matrix [4,5]. 

Disintegrins are a family of small cysteine-rich polypeptides which display a series of biological functions such as inhibition of cell adhesion, migration and angiogenesis. Disintegrins are commonly processed from PII metalloproteinase precursors through proteolytic processing and have tripeptide motifs RGD, KGD, WGD, VGD, MGD, RTS, KTS among others, which confers different binding specificities [6,7,8]. Currently, disintegrins can be classified in four groups. The first group of short disintegrins displays 41–51 residues and four disulphide bonds (e.g., echistatin-RGD and obtustatin-KTS), the second group are medium-sized and contain approximately 70 amino acids and six cystine bonds (e.g., barbourin-KGD, flavoviridin-RGD and atrolysin E-MVD) while the third group includes long disintegrins with approximately 84-residue polypeptide cross-linked by seven disulphide bridges (e.g., bitistatin-RGD). The fourth group is composed of homodimers or heterodimers with subunits of approximately 67 residues with ten cysteines (e.g., EC3A-VGD) involved in the formation of four intrachain disulphides and two interchain cystine linkages [6,7,8,9]. Of note, disintegrin function and specificity depends on the appropriate pairing of cysteine residues which exposes the tripeptide binding motif that mediates inhibition of platelet aggregation, neutrophil or endothelial cell function [9,10,11,12]. Accordingly, disintegrins have been critical as tools in biochemistry, in the development of anti-platelets agents and potential inhibitors of angiogenesis [13,14]. 

In regard to specificity, one group comprises most of the monomeric disintegrins containing the RGD motif, as well as KGD, MVD, MGD, and WGD. They display inhibitory activity against integrins such as αIIbβ3 (fibrinogen receptor), αvβ3 (vitronectin receptor) and α5β1(fibronectin receptor). Some RGD disintegrins have been reported to interact with α3β1, α6β1, and α7β1, and therefore interfere with cell adhesion to laminin [12]. Alternatively, RGD (jarastatin)- or MLD(EC3)-disintegrins may target αMβ2 or α9β1 in neutrophils, respectively [15]. Another group of MLD motif-containing disintegrins interact with leucocyte α4β1, α4β7, and α9β1 integrins [9]. Finally, KTS and RTS disintegrins are potent and selective inhibitors of α1β1 (receptor for collagen IV) [9,16]. Several updated and in-depth reviews on snake venom disintegrins have recently been published [6,7,8,9,10,11,12,13,14,15,16,17,18,19]. A brief overview of disintegrins targeting platelet and endothelial cells is provided below since these are common targets for venom proteins.

### 1.1. Disintegrins, Platelets and Thrombus Formation

Platelets express three β1 integrins and two β3 integrins: α2β1 (collagen receptor), α5β1 (fibronectin receptor), α6β1 (laminin receptor), αIIbβ3 (fibrinogen receptor), and α_V_β3 (vitronectin receptor). Integrin αIIbβ3 is the most abundant glycoprotein on the platelet surface, with an additional intracellular pool that is exposed on the surface upon activation. It binds several ligands containing an arginine-glycine-aspartic acid (RGD) sequence, such as fibrinogen, fibrin, von Willebrand factor (vWF), fibronectin, thrombospondin, and vitronectin. When activated, integrin αIIbβ3 mediates platelet adhesion, aggregation, and spreading on the exposed ECM of the injured vessel wall as well as thrombus formation by promoting crosslinking between adjacent platelets through its main ligand fibrinogen, or at high shear rates through vWF. In resting platelets, the integrin is in a “low-affinity” state characterized by a highly bent conformation that keeps the binding site for the RGD sequence hidden. On platelet activation, “inside-out” signaling events lead to a conformational switch where integrins undergo complex structural rearrangements and change into a “high-affinity” state, acquiring an extended conformation and being able to expose the RGD binding site. Because many integrins bind adhesive proteins in a RGD-dependent manner, it is possible that additional binding sites may regulate which RGD-containing proteins bind to a particular integrin. The reader is referred to several reviews recently published on integrin properties and functions [1,2,3,20,21].

In some pathologic conditions, platelet activation and coagulation may lead to thrombotic vessel occlusion with obstruction of blood flow and subsequent tissue damage, as in myocardial infarction and ischemic stroke. Understanding the mechanisms of platelet adhesion, activation, and aggregation is important to identify new therapeutic targets for treatment of these severe disabilities. Integrin αIIbβ3 has become an attractive pharmacologic target for prevention of ischemic cardiovascular events due to its importance in platelet aggregation [4,19]. Targeting and inhibiting RGD-dependent integrins may prove to be an effective approach for therapeutic intervention in thrombotic disease. In fact, the first disintegrin was characterized as an antagonist of platelet αIIbβ3 [22]. Accordingly, the structure of disintegrins has been used as a template to design compounds that bind to fibrinogen receptor with higher affinity. For example, Integrilin (eptifibatide) is a synthetic cyclic heptapeptide adapted from the snake venom disintegrin barbourin. Aggrastat (tirofiban) is a non-peptide, tyrosine-derived, RGD mimic synthetic compound originated from echistatin. Both antagonists are competitive inhibitors that bind to the ligand-binding pocket of αIIbβ3, competing with the binding of fibrinogen and vWF. Another strategy to inhibit αIIbβ3 includes the antibody abciximab, a high-affinity antagonist with long half-life (4 h). These three pharmaceuticals were approved for treatment of acute coronary ischemic disease, illustrating the importance of studying exogenous secretions in the development of new therapeutics [23]. 

### 1.2. Disintegrins, Angiogenesis and Cancer

Endothelial cells (EC) express a subset of mammalian integrins including the fibronectin receptors α4β1 and α5β1; collagen receptors α1β1 and α2β1; laminin receptors α3β1, α6β1, and α6β4; vitronectin receptor αvβ3; and osteoponin receptor α9β1. Angiogenesis is characterized by the invasion, migration, and proliferation of smooth muscle and endothelial cells—a process that involves new capillaries to sprout from existing blood vessels. It is a highly regulated process, essential in many physiologic conditions, including development, reproduction, and wound repair. Vascular cell adhesion molecules appear to contribute to its regulation, and several pathologic conditions have been related to unregulated angiogenesis, as in tumor development [1]. Although disintegrins have been characterized as platelet-aggregation inhibitors, several venom disintegrins were also found to block adhesion of human umbilical vein endothelial cells to vitronectin and prevent adhesion of tumor cell lines to ECM components. For instance, Triflavin, a RGD disintegrin from *Trimeresurus flavoviridis* was able to block adhesion and migration of human umbilical vein endothelial cells as well as to inhibit angiogenesis [14]. Contortrostatin, isolated from the venom of *Agkistrodon contortrix contortrix*, is an antagonist of αvβ3 and a potent inhibitor of angiogenesis both *in vitro* and *in vivo* [24]. KTS-disintegrins targeting α1β1 showed a regulatory effect in angiogenesis [9], corroborating the view that collagen receptors are strongly involved in the neovascularization process. Accordingly, studies with obtustatin—a disintegrin from Vipera lebetina obtusa—showed that this inhibitor of α1β1 integrin is effective in blocking FGF-induced angiogenesis in a chicken embryo chorioallantoic membrane assay; it also reduces experimental Lewis lung carcinoma growth in a syngeneic mouse model [25]. Also, lebein-1 inhibits α3β1 integrin-dependent migration and invasion of human MDA-MB-231 breast carcinoma cells towards laminin-511 [26]. In addition, RTS-containing disintegrin jerdostatin inhibits the adhesion of alpha(1)-K562 cells to collagen IV suggesting that it also interferes with α1β1-mediated endothelial cell adhesion to collagen and angiogenesis [16]. 

Although saliva from *Ixodes scapularis* has been reported as a potent inhibitor of angiogenesis [27], only three salivary disintegrins named tabinhibitin [28], tablysin [29] and TabRTS [30] from horseflies have been molecularly characterized as an angiogenesis inhibitor. Therefore, salivary disintegrin inhibitors of angiogenesis remain a relatively unexplored field of investigation.

### 1.3. Disintegrins from Hematophagous Animals

While blood-sucking salivary gland is a major source of antihemostatics such as vasodilators, platelet and coagulation inhibitors [10,31,32,33,34,35], relatively few disintegrins have been molecularly cloned and expressed (Table 1).

**Table 1:** Salivary disintegrins which have been characterized molecularly or functionaly.

Name/reference	Species	Mol wt	IC_50_	R/S/P #	Tripeptide	Cell target	Integrin
**Ticks**							
Variabilin [36]	*D. variabilis*	5	157 nM	N/N/Y	RGD	Platelets	αIIbβ3
ISL929/1373 [37]	*Ixodes* sp.	10	?	Y/N/Y	?	Neutrophils	αMβ2 ?
Monogrin [38]	*A.monolakensis*	10	150 nM	Y/N/Y	RGD	Platelets	αIIβ3
TAI [39]*	*O. moubata*	15	8 nM	N/N/Y	?	Platelets EC	α2β1, α1β1
Disagregin [40]	*O. moubata*	6	104 nM	N/N/Y	RED	Platelets	αIIbβ3
Ixodegrin [41]**	*Ixodes* sp.	7	?	N/N/N	RGD	Platelets?	αIIββ3
Savignygrin [42]	*O. savigny*	7	130 nM	N/N/Y	RGD	Platelets	αIIbβ3
** Horseflies**							
Tabinhibitin [28]	*T. yao*	25	< 40 nM	N/N/Y	RGD	Platelets	αIIbβ3
Tablysin [29]	*T. yao*	25	100 nM	Y/Y/Y	RGD	Platelets EC	αIIbβ3 αvβ3
TabRTS [30]	*T. yao*	25	50 nM	Y/N/Y	RTS	EC	α1β1
** Leeches**							
Decorsin [43]	*M. decora*	4	500 nM	Y/Y/Y	RGD	Platelets	αIIbβ3
Ornatin [44]	*P. ornata*	5	130 nM	Y/N/Y	RGD	Platelets	αIIbβ3
** Worms**							
HPI [45]	*A. caninum*	20	?	Y/N/N	KGD	Platelets?	αIIbβ3?
NIF [46]	*A. caninum*	41	<10 nM	Y/N/Y	?	Neutrophil	αMβ2

# R, obtained in recombinant form; S, structure available; P, inhibition of cell function tested with recombinant or purified proteins. Mol wt, molecular weight (approximate); EC, endothelial cell; NIF, neutrophil inhibitory factor; HPI, hookworm platelet inhibitor; TAI, tick adhesion inhibitor. * TAI has not been molecularly identified. ** Ixodegrin has not been expressed or purified; ? IC_50_, or integrin specificity unknown, or not confirmed.

#### 1.3.1. Ticks

Hard ticks are the most important source of disintegrins among arthropods. This is likely because they must inhibit the interaction of other cell types with ECM components during the prolonged feeding period as part of the mechanism by which they keep blood flowing through its proboscis [35]. Blockade of platelet and endothelial cells integrins also contribute to prevent granulation tissue and wound healing response to an injury.

##### 1.3.1.1. Variabilin

This protein is present in the SGs of the hard tick *Dermacentor variabilis* and inhibits platelet aggregation induced by ADP (IC_50_ ~ 150 nM), collagen, and thrombin receptor peptide SFLLRNP. It also blocks platelet adhesion to fibrinogen. Variabilin is a 4-cysteine, 5-kDa disintegrin containing an RGD motif, but the primary sequence shows little homology to most disintegrins except Ixodegrin from *Ixodes scapularis*. Differing from other RGD-containing proteins, the RGD sequence in variabilin is not located in a loop flanked by cysteines. It is a potent antagonist of the fibrinogen receptor integrin αIIbβ3 and the vitronectin receptor αvβ3 [36].

##### 1.3.1.2. Disagregin

Disagregin is a 6-kDa protein from the SGs of *Ornithodoros moubata* that potently blocks ADP-induced platelet aggregation (IC_50_ 150 nM) [40,47]. It lacks the RGD sequence but displays RED motif in the cysteine-stabilized loop important to present the tripeptide motif to integrins. Disagregin has significant sequence similarity and identical cysteine spacing to disintegrins from other soft ticks such as savignygrin and monogrin, which exhibits a bovine pancreatic trypsin inhibitor (BPTI)-Kunitz folding. In addition, disagregin inhibits platelet aggregation by different agonists, blocks platelet adhesion to fibrinogen, binds to resting and ADP-activated platelets, and also binds integrin αIIbβ3 in activated platelets with *K_D_* ~ 40 nM. Crosslinking experiments also demonstrated binding of disagregin to integrin αIIbβ3. In contrast, disagregin does not affect endothelial cell adhesion to vitronectin, which is mediated by integrin αvβ3 [40,47].

##### 1.3.1.3. Savignygrin

Savignygrin is a platelet aggregation inhibitor purified from the soft tick *Ornithodoros savignyi* and is similar to disagregin. It contains a RGD integrin recognition motif and inhibits platelet aggregation induced by ADP (IC_50_ 130 nM), collagen, thrombin receptor-activating peptide, and epinephrine. It also blocks binding of α-CD41 to platelets, binding of αIIbβ3 to fibrinogen, and adhesion of platelets to fibrinogen, suggesting it targets the fibrinogen receptor. Savignygrin forms a complex with both αIIbβ3 subunits, and this complex formation is unaffected by the activation state. This disintegrin belongs to the BPTI family of serine protease inhibitors and presents the integrin RGD-recognition motif on the substrate-binding loop of the Kunitz fold [42]. Additionally, savignygrin can promote disaggregation—which is an inhibition of platelet aggregation at a post aggregation level—through occupation of the αIIbβ3 receptor. Savignygrin-like molecules have also been cloned from the soft tick *Ornithodoros coriaceus* [48].

##### 1.3.1.4. Monogrin

Monogrin was purified from the SGs of the soft tick *Argas monolakensis*. It is a 10-kDa protein containing an RGD motif and having sequence homology to savignygrin and disagregin. It also presents the RGD integrin-recognition sequence on the substrate-binding loop of the Kunitz/BPTI-domain. Both recombinant and purified monogrins block ADP-induced platelet aggregation (IC_50_ ~ 150 nM) but not initiation of shape change. Monogrins were found to interact with integrin αIIbβ3 by surface plasmon resonance [38].

##### 1.3.1.5. Ixodegrin

This family was named after identification of *Ixodes pacificus* [41] and *I. scapularis* putative cysteine-rich proteins with an RGD or KGD domain indicative of proteins that interfere with fibrinogen binding to platelets, acting as platelet aggregation inhibitors. Ixodegrins display sequence similarity to variabilin. The ixodegrin family I (exclusive of the genus Ixodes) was shown to be similar to the short neurotoxin family found in elapid snakes. Many members of the ixodegrin family contain a prokineticin motif, mostly due to the conserved cysteine framework [35,41]. Recently, a protein described from the SGs of the tick A*mblyomma variegatum* showed similarities to *I. scapularis* ixodegrins but does not have the RGD domain [49]. Ixodegrin remains to be produced in a heterologous system to confirm its functional activity.

##### 1.3.1.6. Tick Antiplatelet Inhibitor (TAI)

TAI (~15 kDa) has been purified from *O. moubata* SGs but has not been molecularly cloned. It inhibits platelet adhesion to soluble collagen under static conditions (IC_50_ 8 nM) without affecting the onset or maximum aggregation triggered by collagen or other platelet agonists. TAI also affects endothelial cell adhesion to collagen and has partial inhibitory activity for fibronectin-mediated platelet adhesion. Further, it outcompetes anti-α2β1 monoclonal antibody Gi9 binding to platelets, suggesting it is an integrin α2β1 antagonist [39].

##### 1.3.1.7. ISL929/1373

Two *Ixodes scapularis* salivary proteins named ISL929 and ISL1373 have been described as neutrophil inhibitors. Expression of both molecules is induced upon tick feeding and mostly expressed in the salivary gland. Recombinant ISL929 and ISL1373 appear to reduce expression of β2 integrins, and to decrease production of superoxide by neutrophils *in vitro*. Furthermore, mice immunized with both proteins had increased number of neutrophils at the site of attachment suggesting that they interfere with inflammation *in vivo* [37]. It remains to be demonstrated whether ISL929/ISL1373 targets αMβ2, and whether an inhibitory tripeptide motif is responsible for this activity.

#### 1.3.2. Tabanids

The mouthparts of tabanids operate as “scissors” to cut the skin, leading to formation of a pool of blood from which they feed. While they are considered fast feeders, it is likely that addition of disintegrins to their salivary repertoire has evolved to successfully prevent platelet aggregation or endothelial cell function.

##### 1.3.2.1. Tabinhibitin

Five platelet aggregation inhibitors (tabinhibitin 3–7) were purified from *T. yao Macquart* salivary gland and the cDNA sequences cloned from a cDNA library. Another three cDNA sequences code for tabinhibitins 8–10. These proteins have ~22–25 kDa and display 8–12 half-cystines. There are one or two Arg-Gly-Asp (RGD) motifs in their sequences. Most of the RGD motifs are in the *N*-terminus of their sequences, whereas a RGD motif is in the *C*-terminus of tabinhibitin 3 and 4. All the RGD motifs are positioned in a loop bracketed by cysteine residues as found in other platelet aggregation inhibitors. Members of this family effectively block platelet aggregation by a number of agonists [28]. 

##### 1.3.2.2. TabRTS

A protein from the antigen 5 family containing a RTS disintegrin domain was characterized from the SGs of the horse fly *Tabanus yao* and named tabRTS. The RTS sequence is positioned in the *C*-terminus in a loop flanked by cysteine residues as reported for snake venoms disintegrins, although they do not share any sequence similarity. TabRTS was shown to inhibit endothelial cell proliferation and angiogenesis *in vitro* and *in vivo*; it possibly targets the α1β1 integrin, as anti-α1β1 monoclonal antibody dose-dependently inhibits its anti-angiogenic activity [30].

##### 1.3.2.3. Tablysin-15

Another protein characterized from the tabanid *T. yao* is tablysin-15, a 26-kDa disintegrin containing 10 cysteines and an RGD in the *N*-terminus [29]. Tablysin displays a strong sequence homology to members of the Tabinhibitin family [28], but not to other disintegrins. Tablysin-15 displays a high affinity for αIIbβ3, inhibiting platelet aggregation induced by collagen, ADP, and convulxin. It also blocks thrombus formation under flow at high shear without interfering with platelet adhesion to collagen. Tablysin also inhibits platelet adhesion to fibrinogen under static conditions. When immobilized in solid phase assays, it supports platelet adhesion by a mechanism that is blocked by anti-integrin αIIbβ3 antibody abciximab. Furthermore, it prevents binding of anti-integrin αIIbβ3 antibody to platelets. Notably, tablysin-15 also interacts with endothelial cell αvβ3 integrins, as it prevents EC adhesion to vitronectin with IC_50_ in the nanomolar range. It also affects endothelial cell adhesion to fibronectin at high concentrations (μM range), but does not interfere with endothelial cells adhesion to collagen. Tablysin-15 effectively prevents thrombus formation *in vivo* in the rat arteriovenous shunt thrombosis model [29]. Finally, the structure of tablysin has been recently solved and found to display a pocket adapted to bind leukotrienes (LT) and to inhibit LTC4-induced contraction of a preparation of guinea pig ileum [50]. 

#### 1.3.3. Leeches

Leeches are highly specialized animals that feed on blood for prolonged periods of time. The need to counteract host response to damage has contributed to the development of a notable arsenal of antihemostatics, and particularly disintegrin, as described below.

##### 1.3.3.1. Decorsin

This 39-aa protein purified from the North American leech *Macrobdella decora* acts as an antagonist of glycoprotein GPIIb-IIIa. It completely inhibits platelet aggregation induced by ADP at high concentrations (1 µM) and inhibits the interaction of GPIIb-IIIa with fibrinogen in a solid-phase ELISA (IC_50_ ~ 1.5 nM). Decorsin has 6 cysteines and an RGD motif near its *C*-terminus, which is the significant region of homology to the snake family of inhibitors. Its function is likely to keep host blood flowing or to keep ingested blood from coagulating, as leeches store ingested blood for long periods of time [43]. Recently, decorsin was also described in the salivary transcriptome of *M. decora* [51]. The structure of decorsin was determined by nuclear magnetic resonance and is similar to that of hirudin, an anticoagulant that inhibits thrombin from the leech *Hirudo medicinalis* [52].

##### 1.3.3.2. Ornatin

Six isoforms of ornatin were purified from the leech *Placobdella ornata* and show 40% similarity to decorsin. The purified and recombinant ornatins contain an RGD sequence as well as 6 cysteine residues. Ornatin potently inhibits fibrinogen binding to integrin αIIbβ3 (IC_50_ ~ 5 nM) but inhibits platelet aggregation at higher concentrations (IC_50_ ~ 300 nM) [44]. Studies with recombinant ornatin suggested that the RGD conformation is determined by the disulfide bonds in the native structure, which is important for binding affinity to αIIbβ3 and, consequently, for antagonism of fibrinogen binding [53].

#### 1.3.4. Worms

The chronic survival of many endoparasites—particularly at the mucosa of the host—has been an important evolutionary pressure for the expression of disintegrins, which assist worms to feed on blood. Only two disintegrins from worms have been reported.

##### 1.3.4.1. Hookworm Platelet Inhibitor (HPI)

HPI is a KGD-containing molecule purified from the hookworm *Ancylostoma caninum*. Purified HPI blocks fibrinogen binding to αIIbβ3, or epithelial cell adhesion to collagen which is mediated by integrin α2β1. However, recombinant HPI expressed in *Escherichia coli* does not inhibit integrin binding activity [45]; it is therefore unclear whether recombinant HPI was correctly folded.

##### 1.3.4.2. Neutrophil Inhibitory Factor (NIF)

Neutrophil inhibitory factor (NIF) is a well-studied protein of 41-kDa isolated from canine hookworm *Ancylostoma caninum*, which was found to interact with neutrophil αMβ2 integrin (MAC-1) [46]. NIF inhibits neutrophil adhesion to endothelial cells and inhibits formyl methionyl leucyl phenylalanine (fMLP)-dependent adhesion of PMN to ICAM-1 and the release of H_2_O_2 _by PMN [46,54,55]. NIF binds with high affinity to the metal ion-dependent adhesion site on the A domain of the CD11b subunit of PMN [46,54,55,56]. A functional tripeptide in NIF interacting with αMβ2 integrins has not been identified. 

### 1.4. Construction and Content: Identification of Putative Disintegrins

**Figure 1:**
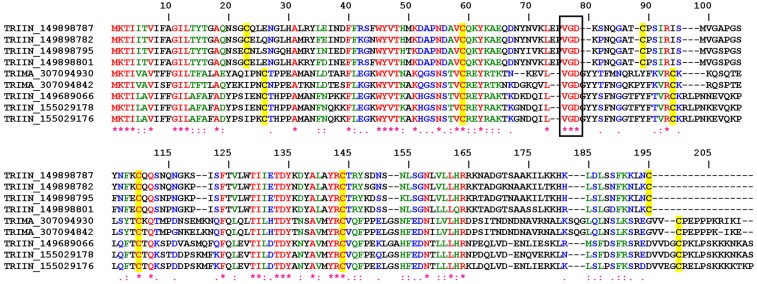
ClustalW alignment for putative VGD disintegrins from Triatominae.

**Figure 2:**
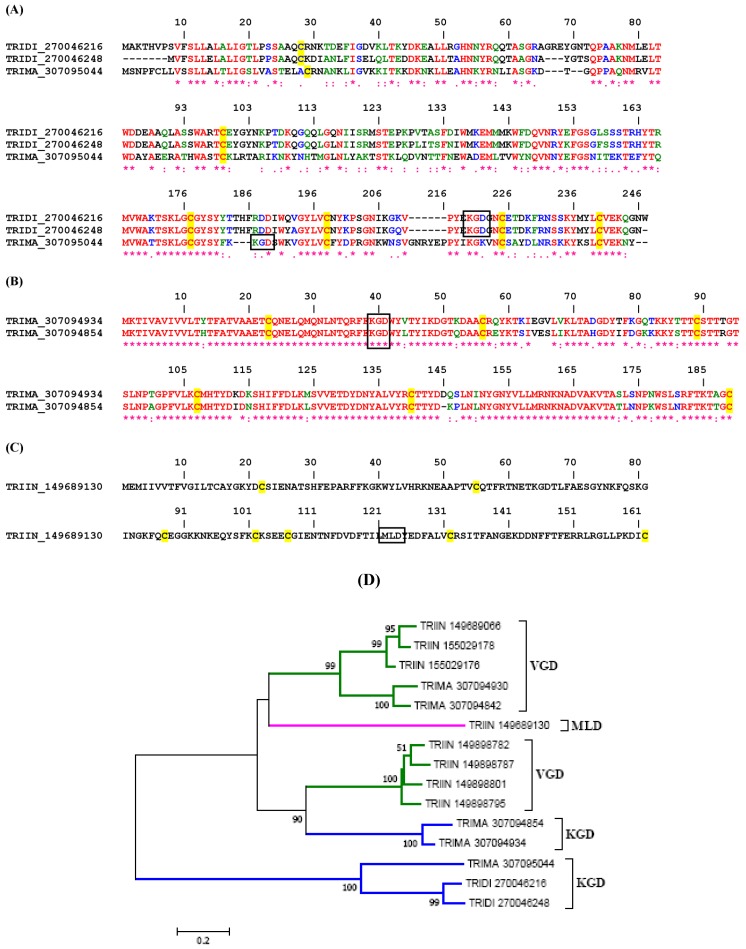
Clustal alignment for putative KGD (**A** and **B**) and MLD (**C**) disintegrins from Triatominae. (**D**) Neighbor-joining phylogram for the sequences presented in A–C, and Figure 1. The numbers in the phylogram nodes indicate percent bootstrap support for the phylogeny. The bar at the bottom indicates 20% amino acid divergence in the sequences.

**Figure 3:**
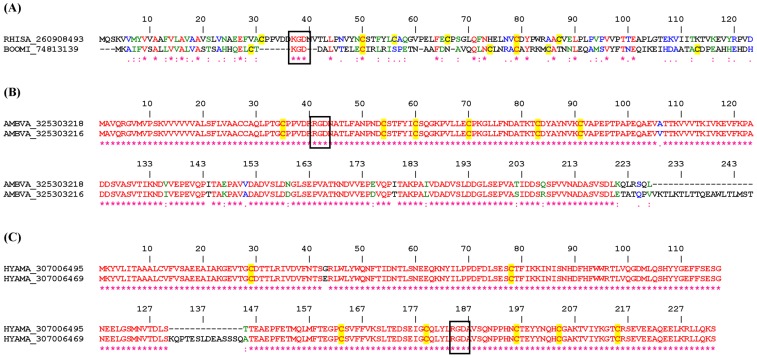
ClustalW alignment for putative KGD (**A**), and RGD (**B** and **C**) disintegrins from Metastriate ticks.

**Figure 4:**
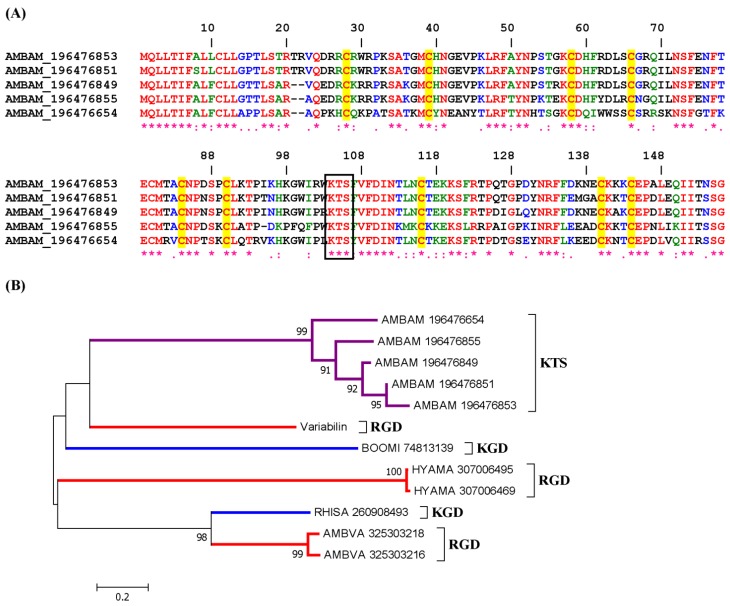
ClustalW alignment for putative KTS (**A**) disintegrins from *Amblyomma* sp. Ticks;(**B**) phylogenetic tree for the sequences presented in A and from other KGD or RGD disintegrins from other metastriates (alignment not shown).

**Figure 5:**
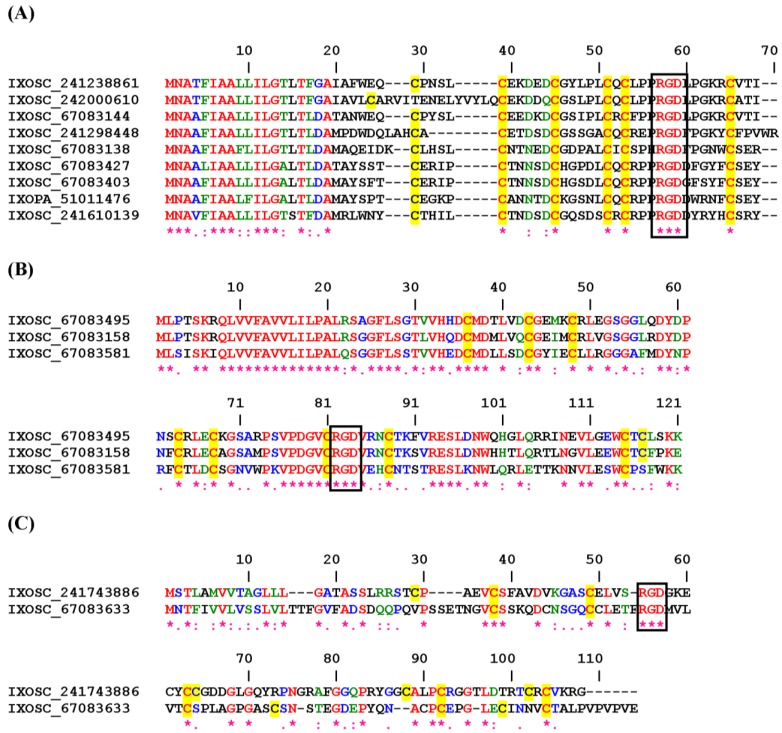
ClustalW alignment for putative RGD (**A**), and RGD (**B** and **C**) disintegrins from Ixodidae ticks.

**Figure 6:**
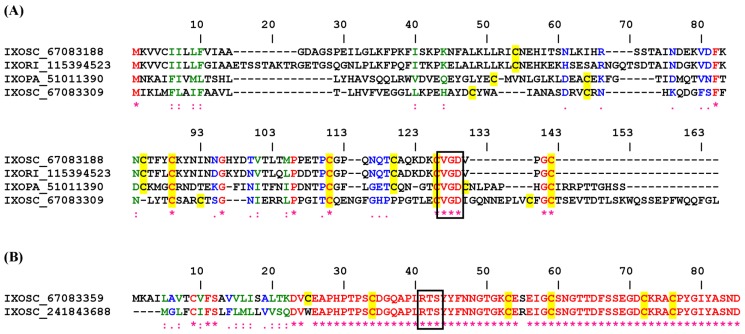
ClustalW alignment for putative VGD (**A**) and RTS (**B**) disintegrins from Ixodidae ticks.

**Figure 7:**
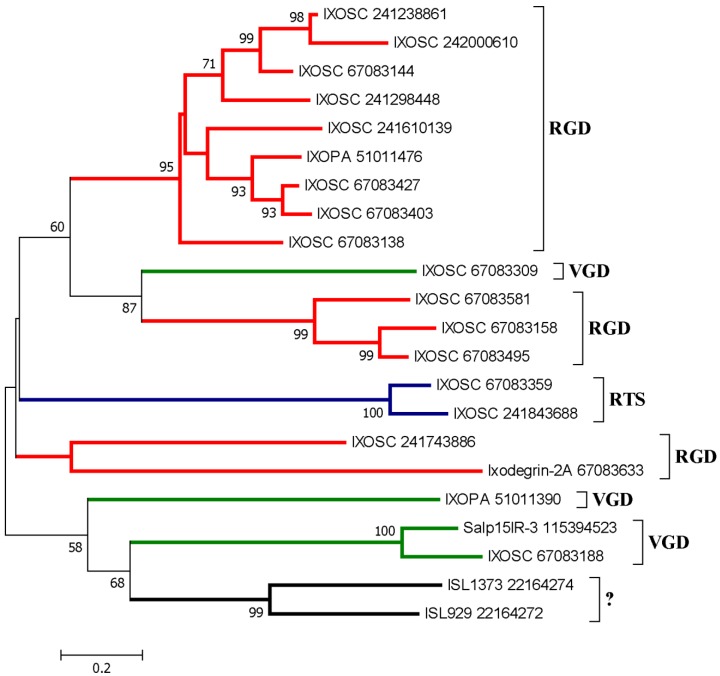
Neighbor-joining phylogram for the Ixodidae sequences presented in Figure 5 and Figure 6. The numbers in the phylogram nodes indicate percent bootstrap support for the phylogeny. The bar at the bottom indicates 20% amino acid divergence in the sequences.

**Figure 8:**
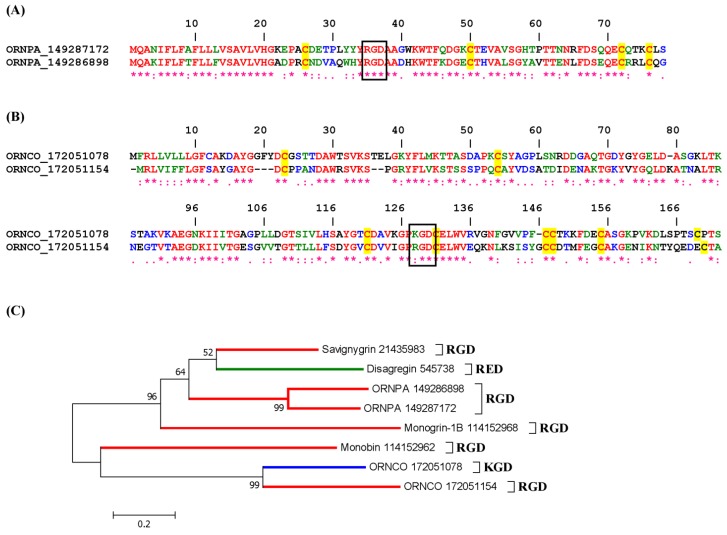
ClustalW alignment for putative RGD (**A**) and K/RGD disintegrins; (**B**) from *Ornithodoros* ticks. **C**, Neighbor-joining phylogram for the Ixodidae sequences presented in (**A**) and (**B**). The numbers in the phylogram nodes indicate percent bootstrap support for the phylogeny. The bar at the bottom indicates 20% amino acid divergence in the sequences. The sequences of other known disintegrins (e.g., disagregin, savignygrin, monogrin) in addition to monobin (a thrombin inhibitor with RGD) [38] have also been included.

**Figure 9:**
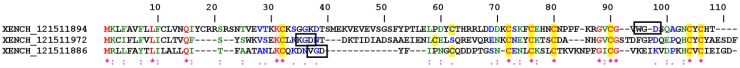
ClustalW alignment for putative WGD, KGD and VGD disintegrins from the rat flea *Xenopsylla cheopis*.

To expand our understanding of the complexity of disintegrin in different species of bloodsucking animals, we have created an algorithm defined as C-x(0,16)-X-Y-Z-(0,16)x-C to search novel putative disintegrins which have been deposited in the non-redundant database. This algorithm was constructed based on the sequences of several disintegrins characterized thus far, which in most cases display the tripeptide motif (e.g., RGD) flanked by cysteines residues critical for exposure of disintegrins to different integrins. In the algorithm formula, XYZ can be substituted for one of the following: RGD, MLD, KGD, VGD, KTS, RTS, WGD, and RED. The starting protein set comprised proteins from GenBank downloaded using the selected organism terms: Ixodoidea, Cimicomorpha, Tabanidae, Glossina, Culicidae, Psychodidae, Simuliidae, Ceratopogonidae, Siphonaptera, Hirudinea and Rhabditida. This set was further reduced by selecting solely those proteins having a signal peptide indicative of secretion as indicated by the SignalP server [57]. Finally, the disintegrin search algorithm was run on these sequences, using the program seedtop that is part of the blast package [58] and bioinformatics tools which have been described elsewhere [59,60,61,62,63,64]. While this approach may identify several novel putative disintegrins, confirmation of their biological activity is imperative in order to verify functionality and to exclude false-positives. Below, we describe novel putative family of disintegrins from hematophagous sources, whose sequences have been deposited in the Genebank as of October 2011. A selected number of representative sequences coding for putative disintegrins are aligned and presented in Figure 1, Figure 2, Figure 3, Figure 4, Figure 5, Figure 6, Figure 7, Figure 8, Figure 9, while several others are displayed in the Supplemental data. Supplemental Table presents all sialogenins containing disintegrin motifs (e.g., RGD) which have been sequenced thus far. The sequences are freely available for download with several relevant hyperlinks at http://exon.niaid.nih.gov/transcriptome/RGD/RGD-sialogenins.zip (stand alone) and http://exon.niaid.nih.gov/transcriptome/RGD/RGD-Peps-WEB.xlsx (web version).

## 2. Discussion

A database for salivary disintegrins appears to be particularly useful since no comprehensive database has yet been published for known salivary disintegrins, or putative ones. Therefore, organizing the database as tables, and providing the information for each disintegrin in excel spread sheets which are fully available to the community may advance our understanding over other available resources. In addition, the finding that several novel sequences were found to display disintegrin motifs lead us to describe and discuss their putative function and target in more detail, based on the tripeptide motifs they display. As discussed before, all putative disintegrins described herein awaits confirmation for their function and specificity.

### 2.1. Disintegrins from Bugs (*Triatoma* and *Rhodnius* sp.)

#### 2.1.1. VGD Disintegrin Family

Figure 1 shows the Clustal alignment for nine molecules which typically display 6 cysteines, a VGD tripeptide in position 76 (flanked by cysteines) and high degree of conservation among other amino acids. Two subfamilies of this family were recognized in *T. infestans*; in one the *C*-terminus ends in a cysteine residue, while in the other a cluster of lysines (K) is found suggesting that these members evolved to interact with activated membranes enriched in phosphatidyl serine. The counterpart of this subfamily in *T. matogrossensis* displays a cluster of prolines in the *C*-terminus, whose function is currently unknown. Since VGD disintegrins have been reported to interact with α5 integrins, and since this is the receptor for fibronectin in the endothelial cells, it is possible that these putative disintegrins are associated with inhibition of angiogenesis or modulation of endothelial cell function. 

#### 2.1.2. KGD, RGD and MLD Disintegrin Family

Figure 2A depicts the alignment for KGD disintegrins from *Triatoma* sp. They belong to the antigen 5 family of proteins. While members of this family display a high degree of similarity throughout the molecules, including highly conserved 6 cysteines, the position of the KGD is curiously distinct. For example, the KGD in two members of *T. dimidiata* proteins is found between cysteines 4 and 5, while in one member from *T. infestans* the KGD is located between cysteines 3 and 4. The function of these proteins is likely involved with blockage of β3 integrin leading to inhibition of platelet integrin αIIbβ3. In fact, one antigen 5 family member (e.g., tablysin-15) has been recently described as a platelet and angiogenesis inhibitor through interaction with αIIbβ3 and αvβ3 [29], and to bind leukotrienes [50]. 

Figure 2B depicts two additional triatomine lipocalins which display the KGD motif at the *N*-terminus of the molecule. Figure 2C presents another putative lipocalin disintegrin from *Triatoma infestans*, which uniquely displays a MLD motif. While this is not an abundant transcript according to sequencing of *T. infestans* cDNA library, its function is possibly involved with β1 integrins. A phylogenetic tree with putative disintegrins from Triatominae is displayed in Figure 2D.

### 2.2. Disintegrins from Metastriate Ticks (Dermacentor, Rhipicephalus, and Amblyomma).

#### 2.2.1. KGD and RGD Disintegrin Family

Figure 3A displays two distinct KGD disintegrins from the salivary glands of *Rhipicephalus* and *Boophilus* (currently classified as *Rhipicephalus*) ticks; both have been identified as mucin-like proteins. Figure 3B shows the alignment of RGD-disintegrins from *Amblyomma* sp. which have been identified as chitin-binding peritrophin (midgut protein) and Figure 3C displays 2 sequences from *Haemaphysalis* sp. salivary gland which belong to the lipocalin family, one of them having an insertion between amino acids 130 and 145. Perhaps the RGD in these proteins is adapted for integrin recognition.

Supplemental Figure 1 reports on 2 members from *Dermacentor* sp. salivary gland which are highly related, including 14 cysteines, and a KGD found between cysteines 10 and 11. Interestingly, a shorter sequence from *R. appendiculatus* was found to display high degree of similarity to the other two members from *Dermacentor* sp.; the KGD is also located between two cysteines; perhaps these molecules have evolved to interact with β3 integrins.

Interestingly, these proteins belong to the lipocalin superfamily of proteins, which in many cases are binders of small ligands such as biogenic amines, prostaglandins and leukotrienes [65]. Other lipocalins have evolved to interact with macromolecules (e.g., thrombin) [66]. Members of this family have been named triafestins (TRIIN_155029178 and TRIIN_155029176), and have been identified as inhibitors of the kallikrein-kinin system [67]. It is not known whether these proteins also display disintegrin properties.

#### 2.2.2. KTS/RTS Disintegrin Family

A family of KTS disintegrin was found in *Amblyomma americanum* salivary gland, and the Clustal alignment is shown in Figure 4A. The phylogenetic tree shows a clade with KTS-containing sequences from *Amblyomma* sp. with a strong bootstrap support, but apart from other genus of metastriates containing RGD or KGD (Figure 4B). Notably, these proteins belong to the Kunitz family of protein inhibitors. These are abundant transcripts coding for putative disintegrins with which the KTS tripeptide appears to be properly placed to interact with α1β1 integrins, assuming the specificity is the same as reported before for the viperidae KTS [9]. It is notable that these inhibitors were found in *Amblyomma* ticks, which remains attached for days in the host. It is possible that these molecules contribute to block endothelial cell adhesion to collagen and to assist in the inhibition of angiogenesis and host response to injury [27]. Supplemental Figure 2 and Figure 3 show three other molecules with KTS or RTS motifs, respectively, found in *Amblyomma* or *Rhipicephalus* sp. It is possible that these molecules interact with α1β1 integrins.

#### 2.2.3. Duodegrins

Bioinformatic analysis identified several sequences with more than one tripeptide motif. These sequences are herein named duodegrins. In some proteins, we found VGD and RTS, while in others we identified RED and VGD. These sequences code for cysteine-rich proteins of high molecular weight in the midgut of ticks, and include the protein named BM86 which is used as a vaccine against tick infestation [68,69]. While its function is unknown it might be related to protection of the tick gut against host neutrophil attack. It is also unknown whether any of these proteins behave as disintegrins. Alignment is reported in Supplemental Figure 4.

### 2.3. Disintegrins from Prostriate Ticks (*Ixodes* sp.).

#### 2.3.1. RGD, KGD, and VGD Disintegrin Family

Figure 5A shows the Clustal alignment of short proteins from *Ixodes* sp. which displays a typical RGD flanked by cysteines 5 and 6. These proteins have no match to other proteins deposited in the database, and have been classified as putative secreted salivary proteins. Likewise, a second family of putative RGD secreted sialogenins is presented in Figure 5B. In Figure 5C, two related putative disintegrins were aligned, and one of them (IXOSC_67083633) has been previously named ixodegrin-2A [41]. It is possible that these are platelet inhibitors. Finally, three other sequences with the KGD motif were discovered in ixodid ticks (Supplemental Figure 5).

Figure 6A reports 4 disintegrins from *Ixodidae* sp. ticks with VGD motif in their *C*-terminus. One of them has been identified as SALP15IR-3 precursor, known to operate as a immunosupressor through interactions with the T cell co-receptor CD4 [70]. It remains to be demonstrated whether the VGD motif mediates interaction of SALP15IR-3 with its receptors, or whether it interacts with α5 integrins (a usual target for VGD disintegrins).

#### 2.3.2. RTS Disintegrin Family

One sequence was found to display RTS motif properly flanked by cysteines, suggesting that this molecule might work as a disintegrin targeting α1β1 (Figure 6B). This is a putative secreted protein without database hits. As reported for other RTS disintegrins, this sequence may also contribute to blockade of angiogenesis by tick saliva [27]. Figure 7 display a phylogenetic tree containing several salivary disintegrins from Ixodidae. It is clear that they clade as different families. Supplemental Figure 6 and Figure 7 show additional putative disintegrins with KTS or KGD/RGD motifs, respectively.

#### 2.3.3. Disintegrins from *Ornithodorus* sp.

Soft ticks are unique in respect to their feeding behavior since they feed for about 30 min. Therefore, their repertoire of anti-hemostatics differs significantly from hard ticks who feed for several days [35,38,41,48,49,71,72,73]. Figure 8A displays the alignment of two short RGD disintegrins from *O. parkeri*, which have been identified as savignygrin-like-1, and -2 [73]. They are likely platelet aggregation inhibitors. Figure 8B displays other two sequences containing RGD motifs from *O. coriaceus* [48] belonging to the lipocalin family of proteins, and having 8 cysteines. It is unclear whether they target platelets, neutrophils or endothelial cells, until they are obtained in recombinant form. Figure 8C depicts the phylogenetic tree for soft ticks’ salivary disintegrins. Supplemental Figure 8 and Figure 9 show putative soft ticks disintegrins expressing RGD, KTS, RTS, VGD and MLD.

#### 2.3.4. Disintegrins from Fleas

Fleas are blood-sucking animals which, despite the low volume of blood ingested, have developed anti-hemostatics such as an apyrase, according to a transcriptome analysis [61]. Three sequences of proteins from fleas were found to contain disintegrin motifs WGD, KGD and VGD located in distinct parts of the molecule (Figure 9). These molecules also present 8 cysteines in the mature form, suggesting that they might work as inhibitors of cell function through integrin blockade. While these are putative secreted proteins, XENCH_121511972 belongs to the antigen-1 precursor like protein [61]. Their function is unknown.

## 3. Conclusions

Supplemental Table 1 presents all sialogenins presenting disintegrin motifs (e.g., RGD) sequenced thus far from hematophagous sources. At present, it is unknown whether most of them operate as disintegrins, and it is also unknown whether they are monomeric or dimeric. Also, because a distinct pattern of cysteines is observed when snake venom and known salivary disintegrins (e.g., monogrin, tablysin) are compared, it is conceivable that a novel nomenclature will be needed in an attempt to classify disintegrins from hematophagous sources. It is also possible that a number of disintegrins in saliva are devoid of the typical tripeptide motifs studied herein, and were therefore missed by our algorithm. For example, NIF which blocks integrin αMβ2 in neutrophils [46] does not contain any of the typical motifs characterized in venoms, and the same is true for ISL929/1373 [37]. Therefore, Supplemental Table 1 should be considered as a platform for future studies, and not as a definitive database. Hopefully it will be expanded to allow investigators to functionally identify new members of the disintegrin family of proteins in blood-sucking arthropods. This database will also provide candidates for understanding structural features of disintegrins [50,74,75,76]. As a final remark, it is somewhat surprising that with exception of Tabanids, none of the Diptera, including mosquitoes, sand flies and biting midges have putative salivary disintegrins, even though three mosquito genomes have been sequenced, indicating that the motif is not at all promiscuous in nature. Alternatively, novel motifs await identification in salivary disintegrins. 

## Supplementary Files and Correction

Supplementary File 1: PDF-Document (PDF, 67 KB)

Supplementary File 2: XLS-Document (XLS, 32752 KB)
