# Supplementary material for: Disintegrins from Hematophagous Sources
Source: Toxins (Basel). 2012 Apr 26;4(5):296–322. doi: 10.3390/toxins4050296 (PMC3386632; doi:10.3390/toxins4050296)
Supplement: Supplementary File 1: — PDF-Document (PDF, 67 KB) [file toxins-04-00296-s001.pdf]

(A)

```
                          10        20        30        40        50        60        70        80        90
                           |         |         |         |         |         |         |         |         |
DERAN_27466898    MWLLLATLGAAQCLAALAQLGDGVAGPEGGVPPLGDCVCVPYYQCKDGKVSEDGGGLLDARRKLPPKEEIPLDGLGDRNQQCPGVDQVCCAEPSAVTE
DERVA_26420454    MWLLLATLGAAQCLAALAQ-GDGVAGPEGGVPPLGDCVCVPYYQCKDGKVSEDGGGLLDARRKLPPKEEIPLDGLGDRNQQCPGVDQVCCAEPSAVTE
RHIAP_18958000    ------------------------------MSPVAGVLAFLAVACTN----------------MGTSAWTNLDGCG---------------------Q
                                                :.*:.. :..    *.:                : ..    *** *                     :

                         108       118       128       138       148       158       168       178       188
                           |         |         |         |         |         |         |         |         |
DERAN_27466898    TPYVASCGIRNDNGINSRILTKDGKGEAEFGEWPWQAAVLKYESEILKFECGGTLIASRYVLTVAHCVARFTGADRVPLKVRLGEWDTQSMKEFYPHE
DERVA_26420454    TPYVASCGIRNDNGINSRILTKDGKGEAEFGEWPWQAAVLKYESEILKFECGGTLIASRYVLTVAHCVARFTGADRVPLKVRLGEWDTQSMKEFYPHE
RHIAP_18958000    TPIAPQLENRED-----R--VVDGQ-EAVPGSWPWHAGLHSSPFFESAYFCGGALISDRHVLTAAHCLEHHAAAN-TFVHVGSHSPCSRDVTEQY---
                  ** ...   *:*     *  . **: **  *.***:*.: .       : ***:**:.*:***.***: :.:.*: . ::*   .  ::.:.* *

                         206       216       226       236       246       256       266       276       286
                           |         |         |         |         |         |         |         |         |
DERAN_27466898    DYDVGNIYIHQYFRNSSLWNDIALLELTRPVSFAPHISPICLP-KLEDVFEGSSCVVTGWGKDAYRTGKFANIMKEVTVPVIDNPTCQNLLRQTRLGR
DERVA_26420454    DYDVGNIYIHQYFRNSSLWNDIALLELTRPVSFAPHISPICLP-KLEDAFEGSSCVVTGWGKDAYRTGKFANIMKEVTVPVIDNPTCQNLLRQTRLGR
RHIAP_18958000    ---VGAEHLCMHVDPE---RDIGIVKLKSSVNFTDTVRPACLPEPGSELADGTTLYVTGWGQTDETD--------PSSR-ADGCSSLTKSIANERCAK
                     **  ::  :.  .   .**.:::*. .*.*:  : * ***   .:  :*::  *****:               :  . . .:  : : : * .:

                         304       314       324       334       344       354       364       374       384
                           |         |         |         |         |         |         |         |         |
DERAN_27466898    YFRLHEGFICAGTEDGVDSCKGDGGGPLSCYAADGRYHLAGLVAWG-IDCGTPDVPGV-YMRVAKYLDWISEVTHLPIS-------EFYPK-------
DERVA_26420454    YFRLHEGFICAGTEDGVDSCKGDGGGPLSCYAADGRYHLAGLVAWG-IDCGTPDVPGV-YMRVAKYLDWISEVTRLPIS-------EFYPK-------
RHIAP_18958000    EFHEVPDYLLCGSYDYGTPCQGDSGGPLARKGDDGAWVLEGIVHKGGQLCKSFNFRAMRYMKVSHFVNWIDDYMRADAEGRADSFCDMAPNFKLDRGV
                   *:   .:: .*: *   .*:**.****:  .  **  : * *:*  *   *  * : :. .: **:*:::::**.:  :   .       :: *:
```

(B)

```
                          10        20        30        40        50        60        70
                           |         |         |         |         |         |         |
HAELO_68264899    MRTSLLVFVICYISYGTGSLPIECQRKFRTATGNCNNEGPSVRYGYDKEKGDCVRYYYNSCRGNKNNFASRSECL

                          85        95       105       115       125       135       145
                           |         |         |         |         |         |         |
HAELO_68264899    NRCNPESRCLLFTYENEGNWRLFKSYYYNATLDECRLTKTYTYHSTSEKYNRFANMKDCEKACRRNDTEDVYSSG
```

(C)

```
                          10        20        30        40        50        60        70        80        90       100
                           |         |         |         |         |         |         |         |         |         |
AMBVA_325302602   MNCLRIGLLFMAIFLAEAQRTATRNQCSYIERRSTWPRYDQFCKPNLTPRWELYKPRLCLCKQGYIRNSWGHCIKRQECNSCRHVRKKDFKQCSSACPLVCGK

                         113       123       133       143       153       163       173       183       193       203
                           |         |         |         |         |         |         |         |         |         |
AMBVA_325302602   RVPQVCTEQCVVGCACPPGYVIHPSNQKSCVPVSHCPPRCPRHSTFQLCSSTCAPSCDSPRPKRCEIRCHEGECVCHPWFATALVKGDQKCVPRNRCPTRGE
```

Figure 1-S – metastriate – KGD (midgut serine proteinase)

```
                           10        20        30        40        50        60        70        80        90
                            |         |         |         |         |         |         |         |         |
AMBVA_325303714    MAAAMMADRLRGLLLG-------------------LLIAVAS----------VCESAP-----PVSQRAEHGITTQSTAS------GGCSARV
AMBVA_325302736    MQPAVFA-LLLGLVVGDHAVEQTGGHHQRVNHSAGFTIRVSGNNTHSGNSTAWCFEEPE--TGPCRAAMQKWFYNKTSKRCEKFLYGGCSLTT
RHISA_260908261    MRVFLLTFFAYCDVIF--------------------WVPVDAQKGKS-----VCYLMPSEEDDNCDTDVSKWYYNREETNCVNFMYGECPAGN
                   *   :::      ::                      : * .           *   *           .:   .:          * *.

                          103       113       123       133       143       153       163       173       183
                            |         |         |         |         |         |         |         |         |
AMBVA_325303714    RAHQRKTSCR-KCRG---GQRNRYHQ----------ATDHARKQRHC----EQRHRD------------------------------------
AMBVA_325302736    NMFNSKNECNVRCRDPVYGECANFNKNVTCRSYPQAKVNFNPETQSCAWYIATCHADGNTFSSTDACYSHCGEFVAN--PCILPINPGT----
RHISA_260908261    NIFKTSQECNAACQGAGSGPPKPQRPSGPTKQPPRHPPSKGRPPWKKPKYPPKGSEEENETGGGSTKGQGGGVWKPSKRPSKRPPEPKWPPQR
                   . .: . .*.  *:.   *     .             .                 :
```

Figure 2-S – metastriate - KTS

```
                          10        20        30        40        50        60        70        80        90
                           |         |         |         |         |         |         |         |         |
RHISA_260908518   ---MIFVMAAVLASSVVELSEASGTNQSCGDP--IQELGGACSNTEVKQRWGYDSSSGKCVKFLSWDCKKN--------RNNFPTVKECLETCN-----
RHISA_260908350   MDKRAFVLILVLSN-IVPLLAWGSLARVYRRPKTTAKTQQDIQPKAFAARSQYQRGAAKGYKVYQPKCYS---------RTSGTCIYPLLCLCRP----
RHIAP_28932710    -MKLLCALALVALGLPFGSAYLGGFGGLGGWGGGLGAIFGPGAYPGFYGLNKRCTSWAAGSTISSGDSRTPPGIGAAERRGTLSPYPLDINTVQDPVTW
                        .:  *  .  .     ..                      .        . .   .. . .. .         * . .     :   .

                         109       119       129       139       149       159       169       179       189
                           |         |         |         |         |         |         |         |         |
RHISA_260908518   --RDSQCLKDPKRFSLPFHETFYFNVNKEKCEKKRTTNKRTSKKNNRFGSEKECMDQCMPK-------NFELITHSMQ---------------------
RHISA_260908350   --RDELGFHR----VTDADKRWYYNNSTGRCEERMAAPNGCNDFHDKEMCERHCYNVTERVRS---FWNASYVQAVLRQ--------------------
RHIAP_28932710    PPHGTRCLRR--RLAGAPRSRTSPNSTGCACPSPHSSAPAIPSPTPTTSSIPSACPYPVPIHSNTEVHKTDVVAATPGGPVLLESGVTGVRPGEPRVVA
                    :.   ::          .    * .   * .  ::     .      .   .              : . :
```

Figure 3-S – metastriate – RTS

```
                            10        20        30        40        50        60        70        80        90       100       110
                             |         |         |         |         |         |         |         |         |         |         |
BOOMI_115063       MRGIALFVAAVSLIVEGT-------AESSICSDFGNEFCRNAECEVVPGAEDDFVCKCPRDNMYFNAAEKQCEYKDTCKTRECSYGRCVESNPSKASCVCEASDDLTLQCK
RHIMI_306415515    MRGIALFVAAVSLIVECT-------AESSICSGFGNEFCRNAECEVVPGAEDDFVCKCPRDNMYFNAAEKQCEYKDTCKTRECSYGRCVESNPSKASCVCEASDDLTLQCK
RHIAN_219553156    MRGIALFVAAVSLIVEGT-------AKSSICSDFGNEFCRNAECEVVPGAEDDFVCKCPRDNMYFNAAEKQCEYKDTCKTRECSYGRCVESNPSKASCVCEASDDLTLQCK
RHIAP_281188148    MRDLALFTAIVSLIGGCA-------AESSICSDFGDQFCRNAVCEVVSGAEDDFVCKCPRDGMYYNAAEKKCEYKDSCKTKECTYGRCVESAPGKTRCGCENVDTLTLSCK
RHISA_124389894    MRSLALFVAAVSLIGGCA-------AQSSVCSDFGNEFCRNAECEVVSGAEHDFVCRCPRDDMYYNAAEKHCEYKSTCKTKECSYGRCTEISPSKAVCACDGVDSLTLRCT
HYAAN_17154958     MCSPPLFVGAILLIVGCAGQVLRAQPTSSVCSDFGKQFCQNAECEVIPGREDDFVCRCPKDDMYYNAAEKQCEYKRTCKTVECSYGNCVQISPGRTDCGCQGVDTLTLKCG
                   * . .**.. : **    :        . **:**.**.:**:** ***:.* *.****:**:*.**:*****:****  :*** **:**.*.:   *.:: * *:   * *** *

                           121       131       141       151       161       171       181       191       201       211       221
                             |         |         |         |         |         |         |         |         |         |         |
BOOMI_115063       IKNDYATDCRNRGGTAKLRTDGFIGATCDCGEWGAMNM-TTRNCVPTTCLRPDLTCKDLCEKNLLQRDSRCCQGWNTANCSAAPPADSYCSPGSPKGPDGQCINACKTKEA
RHIMI_306415515    IKNDYATDCRNRGGTAKLRTDGFIGATCDCGEWGAMNM-TTRNCVPTTCLRPDLTCKDLCEKNLLQRDSRCCQGWNTANCSAAPPADSYCSPGSPKGPDGQCKNACKTKEA
RHIAN_219553156    IKYDYATDCRNRGGTAKLRTDGFIGATCDCGEWGAMNK-TTRNCVPTTCLRPDLTCKDLCEKNLLQRDSRCCQGWNSPKCSAP--ADSYCSPGSPKEPDGQCKDACKTKEA
RHIAP_281188148    IQGWFTDDCREMGGTARLRRGQFPGATCDCGEWGAMDK-IKRKCVPTTCLRPDLTCKYLCENNLLQRDSRCCEGWNSTNCSAAPPDGSYCSPGTLRGPDGKCKDACTVKEG
RHISA_124389894    IQNWYADECRRMGGTAKLRTDGFLGARCDCGEWSAMDR-NKSKRVPTTCLRPDLTCKYLCENDLLEKDSRCCQGWNSANCSAAPEEGSYCSPGHLKGKDGKCQDACSAKEA
HYAAN_17154958     IQEWYANECGRRGGTAVRRTDGFLGARCDCGEWGKMSKGPNGKCVPTTCIRPDLTCKDLCEKNLLGKDTRCCQGWNPTDCSVVPPEDTYCSPGSIKGEDGKCIDACTTKEA
                   *:  :: :*  . ****  *  .  * ** ******. *.    . : *****:******* ***::** :*:***:***...**.    .:*****  :  **:* :**..**.

                           232       242       252       262       272       282       292       302       312       322       332
                             |         |         |         |         |         |         |         |         |         |         |
BOOMI_115063       GFVCKHGCR---STGKAYECTCPSGSTVAEDGITCKSISHTVSCTAEQKQTCRPTEDCRVHKGTVLCECPWNQHLVGDTCISDCVDKKCHEEFMDCGVYMNRQSCYCPWKS
RHIMI_306415515    GFVCKHGCR---STGKAYECTCPSGSTVAEDGITCKSISHTVSCTVEQKQTCRPTEDCRVHKGTVLCECPWNQHLVGDTCISDCVDKKCHEEFMDCGVYINRQSCYCPWKS
RHIAN_219553156    GFVCKHGCR---STDKAYECTCPRGFTVAEDGITCKSIPYTGGCTVEQKQTCRPTENCRVHAGKVLCVCPWNQHLVGDKCIGDCVENKCHEEFTDCGVYMNRQSCYCPWKS
RHIAP_281188148    NFVCEYGCKS--STKKAYECRCTPGFEVAEDGITCKSIPYTGGCTDEQKQTCRPSEDCRVHKGNVTCECPRGQHLVEDECTSECTENKCHEDFTDCGVSTGRQRCYCPWTI
RHISA_124389894    TFVCKHGCKRSSKPMRAYECRCTSDDEVAEDGITCKGIPYTGGCSDEQKKTCRPSENCLLKKEKVVCECPMRQHLVNDECISDCVDDKCHENFMECGVYMWKQNCYCPWRT
HYAAN_17154958     LLLCKDGCIKGQKPGKAYKCICPHGYEIAEDGITCKRVPGIVDCTEEQKAACLPGQQCRVHKENSVCECPSDQQLLDGKCASECVDNRCHENFTDCGVYMNKQGCYCPWTT
                    ::*: **    .. :**:* *. .  :******** :.    .*: *** :* * ::* ::   .   * **  *:*: . *  .:*.:.:***:* :***    :* *****

                           343       353       363       373       383       393       403       413       423       433       443
                             |         |         |         |         |         |         |         |         |         |         |
BOOMI_115063       RKPGPNVNINECLLNEYYYTVSFTPNISFDSDHCKWYEDRVLEAIRTSIGKEVFKVEILNCTQDIKARLIAEKPLSKHVLRKLQACEHPIGEWCMMYPKLLIKKNSATEIE
RHIMI_306415515    RKPGPNVNINGCLLNEYYYTVSFTPNISFDSDHCKWYEDRVLEAIRTSIGKEVFKVEILNCTQDIRARLIAEKPLSNHVLRKLQACEHPIGEWCMMYPKLLIKKNSATEIE
RHIAN_219553156    RKPGPNVNINECLLNEYYYTVSFTPNISLDSDHCDWYEDRVLEAIRTSIGKEVFKVEILNCTQDIKARLIAEKPLSNHVLRKLQACEHPIGEWCMMYPKLLIKKNSATEIE
RHIAP_281188148    RKPKSGAYINQCELNEYYYTVSFTPNITLNSDHCYSYEALVLEAIRTSIGSEVYKVEILNCTQDIKARLIASKPLSKHVLQKLQACEHPVGDLCMLYPKLPIKKNSATEIE
RHISA_124389894    RKPRPNVFINECVLNEYYYTVSFTPNISLNSDSCDSYAARVLDAIRTSIGSEVFKVEILNCTEDIKARLIASKPLSKHVLKRLQACEHPVGDLCMLYPKLPIKKGSATEIE
HYAAN_17154958     RKPPGGVEISRCMLNEYYYTVSFTPNISLNSDHCEWYEKRVLEAMRTAIGVEVFKVEIMNCTQDIMARLIASRPLSNHVLNKLQACEHPVGDFCMLYPKLPIKKGSATGIE
                   ***    .. *. * **************:.:** *  *    **:*:**:** **:****:***:** ***** .:***:*** .:*******:*: **:**** ***.*** **

                           454       464       474       484       494       504       514       524       534       544       554
                             |         |         |         |         |         |         |         |         |         |         |
BOOMI_115063       EENLCDSLLKDQEAAYKGQNKCVKVDNLFWFQCADGYTTTYEMTRGRLRRSVCKAGVSCNENEQSECADKGQIFVYENGKANCQCPPDTKPGEIGCIERTTCNPKEIQECQ
RHIMI_306415515    EENLRDSLLKNQEAAYKGQNKCVKVDNLFWFQCADGYTTTYEMTRGRLRRSVCKAGVSCNENEQLECTNKGQICVYENGKANCQCPPDTRPGEIGCIERTTCNPKEIQECQ
RHIAN_219553156    EENLCDSLLKNQEAAYKGQNKCVKVDNLFWFQCADGYTTTYEMTRGRLRRSVCKAGVSCNENEQSECADKGQIFVYENGKANCQCPPDTKPGEIGCIERTTCNPKEIQECQ
RHIAP_281188148    EENLCDSLLKRQEAAYKGQNKCVKVGNIFWFQCADGYRAVNETTRGRLRRSVCEAGVSCSENEQLECANKGQICVYEENKANCQCPPGTNVGEVGCVARTTCDPKEIRECQ
RHISA_124389894    EENLCDSILKTQENGYKGQNKCVKVDNFFWFQCADGYRAVDEIERGRLRRSVCVAGLSCSDKEQLECVNKGQICVYENEKANCQCPPGTVAGQAGCTARTTCNPKEVRECQ
HYAAN_17154958     EENLCESLLKNQEKAYKGENKCVKVDDFYWFQCADGYRAVRDVTRGRLRRSVCKAGVSCTDKEQLDCANKGQICVFENEKPNCQCPPDTVPGQAGCAARTTCNPKEIRECE
                   **** :*:** **  .***:****** .:::******** :. :  ********* **:** .:** :* .:**** *:*: *.******.*  *: **  ****:***:**:

                           565       575       585       595       605       615       625       635       645       655       665
                             |         |         |         |         |         |         |         |         |         |         |
BOOMI_115063       DKKLECVYKNHKAECECPD-----DHECYREPAKDSCSEEDNGKCQSSGQRCVIENGKAVCKEKSEATTAATTTTKAKDKDPDPGKSSAAAVSATGLLLLLAATSVTAASL
RHIMI_306415515    DKKLECVYKNHKAECKCPD-----DHECSR---------------------------------------------------------------------------------
RHIAN_219553156    DKKLECVYKNHKAECECPD-----DHECYREPAKDSCSEEDNGKCQSSGQRCVMENGKAVCKETSEATTAATTTTKAKNKDPDPGKSSAAAVSATGLLLLLAATSVTAASL
RHIAP_281188148    DQKRECVFRDQKAECKCPEGTVDDGHGCSREPAKDSCSEEDNRKCGSKGQRCVMEKGQPVCKAISDATTAATTTTKAKDKDQDPGKSSAAAVSATGLLLLVAAASIFAA--
RHISA_124389894    DQKRECVYKDQKAECKCPEGTVDDGDGCSAEES---CSEDEIGKCRSKGQRCVMENGKPVCKEISDATTATTTTTKAKDKNQNPGKSSAAAISATWLLLLIAATSVAAA--
HYAAN_17154958     DKKKECVYRDQKAECQCPEGTVDYGQGCSGGPVEASCTEESIAECRSSGKRCAIENGRPICKETSGVVTAEATTTEATKADPDPGKSGGVAVSATTLLLLAASVAAAAA--
                   *:* ***::::****:**:      .. *
```

Figure 4-S – metastriate – VGD – 2 domains

```
                                 10        20        30        40        50        60        70
                                  |         |         |         |         |         |         |
IXOSC_241690880         MEMKTLGAALALTFAAASLVAFVGAQPVNDDNFK---EVPVGYDEVTPTPREVGQSCNAYSLCKSGLCCLQS-DLSSP
IXOSC_67083399          --MNTFIVVLVSSLALTMFEVFADPDQQPDVTY----SVPSG-------------ICSKNSECGPNLCCRETFKGDMA
IXOSC_241250787         MALYGATPSVCTMALLLLLGLSAVPGGHGDDAFFRRRLVPES------LQTLLGGDGSTQVLGRPEFFKAPREDQAEA
IXOSC_241859419         -MAFSLAGSLGLATTSALLRRTSAAIRCLATSTENIADLVKG-----DKVVVFMKGVPESPRCGFSNAVVQVLRMHGV
                                 :        :     .             :  .

                                 88        98       108       118       128       138       148
                                  |         |         |         |         |         |         |
IXOSC_241690880         ITTCQPRSAPG-----------QRCTGVQVESPFYLDYCPCLKGD---RLFFSPHC----------------------
IXOSC_67083399          VVTCAPLAKSG-----------VPCSNSETGDEPYKTYCSCETG----LECINNVCTALPAPVPVE------------
IXOSC_241250787         VADLPSLMDESQL---------QDCHIEVQVTERLRGHCSTLELM---GKNFPVCRKGDHLSVNHHECSHA-------
IXOSC_241859419         QYKAHDVMEDENLRQGIKAYSNWPTIPQVYIDGQFVGGCDILLQMHQNGELIDELAKVGIKSLLVDAAPASEQETTKK
                                                              *            :
```

Figure 5-S – prostriates - KGD

```
                    10        20        30        40        50        60        70        80
                     |         |         |         |         |         |         |         |
IXOSC_67083629   ----MSGIELILL---LFHICIVYGAETTKCNLTKTSCPGLKEK-WHFNGLIG------------RCERSTPSFCGGKDN
IXOSC_67083208   ----MSGIKLILL---LFHICIVCGVETTQCNLTKTSCPRRKQN-WHFNYLIG------------RCERSTTSFCGGKDI
IXOSC_241802506  ----MNHSTLLLLGAAAFACTIGLCRAGVYKDLAECTTENVEKCGVDFVPYFGGNTL---AVNAADLEKQCTEYLTQLKC
IXOSC_241998684  ---MLRTIAVACL---LLGLFEYTAAGPTTKTPAGCGTESFEKCGTDLIIFAGGPVI---PVSKEELVTTCPKEKTSEKC
IXOSC_241165998  --MKIPGAIIGLAAFVVCAFVLRQGSAASAKCAFPRTTSCFKSHLNDLTGNLTDLTRKQGAALDAALTNYTRRFSAAFQC
IXOSC_242002184  MPSTVHYPAILLCTLSLLAVFLVADSKTLKEGDCEVCVGALKKLHDRLEVEERS--------NEGSVEAGFLEFCKTSKG
IXOSC_241250792  ----LCLGLLLLASSVSAARAGTRPHPGGNQRTLLRPLPSHDLPVIDLVEPAGHVYDP--GPEDLDITRLQRTLAGHFDP
IXOSC_241159543  ---------MSIVKTALLVVLGVVCVSSAFPGVWRKHHPDVDPRYKEWAHFAIS-----------SQVEDRTNFDTLMTL
IXOSC_332691297  -MLTVSLLTLSLAAY-ASAVATVTDANNFMDAVLHTRIPALITSEPILFPFATIP--------PFNFTVAGTNILTNREL
                          :

                    90       100       110       120       130       140       150       160
                     |         |         |         |         |         |         |         |
IXOSC_67083629   TFPDFKECQRTCENAEIITAEDCRMALNKGICEHKKKGRPKKP------------ITRWYFNSTDSNCHKFMWQRCSGNR
IXOSC_67083208   TFSSFEECQRTCEKEEIVSPQDCRMNFNRGTCEEKRKGRPKKW------------VYRWYFNSTDSQCHRFQWYRCSANR
IXOSC_241802506  SDDFSVKCLDGLPKGTILLMLRAARDEYDAICNVTSPRHQEKTSVL----TVCS-LKPFCSFSHGSYYADFANCATSGLK
IXOSC_241998684  ARAYAQKCLARFPRGMVMLLLDGIRTEVNAKCNDSGAGHQG---------------------------------------
IXOSC_241165998  FVESEKRCANSQETQYIGRVQSTSDLLVRELTNKTALTAIVTAYKC----HDVERFTPCMSKAIRDFLSEPLKTSKADAK
IXOSC_242002184  PEHRFCYYVGGLEESATKIVNELTKPFSWGMPALKVCEKLVAK---------------------DSQICDLKYPKVIDLK
IXOSC_241250792  KFMAVHRPKESFLHPNGSLQPGFRLKKGRLVPARPMPRDLQRIRLKGLDFPGGIKVRLDLGRKLRRKLRQLLWTYTYCPL
IXOSC_241159543  ISVESQVIAGVDYKLKMKVAESNCVIGVDSYSRERCHLKVDAP-----------------YMICTALVNYMPWEHKTSLK
IXOSC_332691297  QVNVSRGEIRGFSTEVKRVGDCMPPVLREGKTSIRCTLNFTGIN----ATFDTHTRGDNIVASDKNIWVRASVIDTTGQF

                   170       180       190       200       210       220       230
                     |         |         |         |         |         |         |
IXOSC_67083629   NNFPTKQDCMVCQRAIPTTP----------------TTSTTPATSTTPATPATPAAPAQT--TPPA------------
IXOSC_67083208   NNFPTKEDCQICQKAMPTTP----------------TTSTTPATSTTPATPATPAAPAET--TPPPEC----------
IXOSC_241802506  TKCGDKEAEAFFDTIIQHVFGEVLDL-----ACGKYRAGSEACKSLTPLPTVDDARAKDKGFIEPLTVIASKLG----
IXOSC_241998684  ------------------------------------------------------------------------------
IXOSC_241165998  TLCSNVDDASKTCLVARNCTPAAE-------PGKKAVRKLFTSFRTIWGCKGGSGAPAATSVLPTVSVMLALIARKML
IXOSC_242002184  TVNLKKLKVKDLKKILSDWD-----------------------ER-CEGCVEKTDFVKRIEELKTVHMREEL------
IXOSC_241250792  VYRWKDLGLRFWPRWIREGRCYNSRRSCSFPPGMTCREKTSAEKTVLRWHCRDWTQRRQCRWIPATLSVLVECSCSC-
IXOSC_241159543  SYNCSDRVYGVKAAE---------------------------------------------------------------
IXOSC_332691297  EAVAERGKQGNVHTFLVDKIHVKVKNDKALSLNDKRKKKFRQHFEDKVLTVLPQIFYGAYLHLLGAAVSSVPFPHV--
```

Figure 6-S – prostriates – KTS

```
                            10        20        30        40        50        60        70        80        90       100
                             |         |         |         |         |         |         |         |         |         |
IXOSC_67083443     -------MEIWWCRLFVLVIVAAMQAR--GDPGVPDTPCEDRLPNVATCSEGASEAWYFDLEEKVCRSIIGCNYSTNYFENSTECESVCSPDKYCLLEKPQE
IXOSC_242000850    MGPCYRRSGALFAPLLWLLVGVGSRRRDCGDPVVRTAEARLTSPNYPLAYPAGVRCSYRVLPSWPGVCAVLLSFEDMDIEG--TPPNCERGD---VLRVP-S
IXOSC_241719667    -------MFWLLSQTLLFIDVHGNGVR---------SPLCGGALITPQHVLTAAHCTFNGNKSLTPDAFVARLGEHDYLSN----------DD---------
                               .  : ::   .   *         :         .     . .. :    .    . :    .   :..          *

                          112       122       132       142       152       162       172       182       192       202
                             |         |         |         |         |         |         |         |         |         |
IXOSC_67083443     PVDACVDQEKKTEPNLDNQKNSSLWYFDKKLRKCVETDELSRNTFPSQRHCQASCAKYSVCYAQESAWNQRRCNPELVWYFDPIRKDCFYGKRCGNSTNRFA
IXOSC_242000850    TGDSFCGVTPPESLVLPLPQGGLTLEFSSDTERSGVGFALRLAQVPNSCPTDVTSPCGGRFSEQEFRIEGPSARSGACLYLVTKFRDDVCQLQLR--YDRFS
IXOSC_241719667    ------GANPVDEPVVQIHRHS---DFNSRTYLNDVAVLKLRRPVPLNKDIALICLPYGPLQTDTYEGKMANIAGWGELYYGGPSSASLQDTRIP--IQSLD
                         .     .  :   : .     *..               .*.      .   .    :    :          *        .   :     : :

                          214       224       234       244       254       264       274       284       294       304
                             |         |         |         |         |         |         |         |         |         |
IXOSC_67083443     TREDCLRECPYAGEIPECHLEKDYGSSCPPA----ESGREWGGSWKAFPLARASLRWYYDYVDGECRSFIYQGCGGNENNFPTKRTCNAICRHRTSVITGTA
IXOSC_242000850    ----------VGDNASCTHGRRLIVAGTP-----ECGRKPPGKVDVFEFPSRTL-----------T-LLYLGGDSAHDDSFVLDFFQEECPDTR-------
IXOSC_241719667    T-------CKESFKRTSITFTDNYLCAGSLKGDKDACRGDSGGPLMLLDQQERFT----------IIGITSFGRRCAEPGYPGVYTRVAKYLDWIAQRLN--
                                .  ..         : .       .  .  *    :                    :   *    . .            .
```

Figure 7-S – prostriates – RGD and KGD – 2 domains

```
                          10        20        30        40        50        60        70        80
                           |         |         |         |         |         |         |         |
ARGMO_114152962   ----MRLLALFAFAVAVVSAQR-----NQMCQQPRTQGSCDASNQITKFFY--TGSGCTSAPVCS-DTDGGYGTEDECIQACTVQGG
ORNPA_149287150   -MEAKVFICILVFVLGVSHSTRG---IADKCRKQRQE-CPDKRTGLRRYFFNQTKERCSSFFACPGEGDNFYPMMKDCVKDCRPKQR
ORNCO_172051234   -MDRLAVILTCWLVAGVSESNA-------QCAAQADLDAWDLLDPAGHHTFLLVNSTKSSPRDCLKATAQGNPVKPNAQVELSFKIE
ORNPA_149287112   MTCKFALVVFCLFVVGYVDAASDDLWKLLSASDKFQLISRTYSLGASECAYMKVESRDQSTHTLNTLMGFRDGTTKQYSQPGSFTIT
                        .:    :. .   :          .       .        .  :  . .   *                :

                          97       107       117       127       137       147       157       167
                           |         |         |         |         |         |         |         |
ARGMO_114152962   H--------HNEGAGEEG-CSGDPPRGDCGGQVEERYYFDSTTRTC----QTFEYRGCSSGNPDNSYETEIECEIACPSASS-----
ORNPA_149287150   Q--------PKCFEKELTTCRGDTPGKTAWTHVRKTKQCEEVGNACGDTKNKFASKDECVAECFGFTKTGLQKKYKPKPKKEQG---
ORNCO_172051234   ERWAATNWEFVTNGPKMSATLGDRHEEATIVYGDDTCHVKVLGSGNIEYWKRSDSSNPNPCCQRVFDEKRGDRAFTEPQTKGCTGA-
ORNPA_149287112   VNGDQVTVRRASGSTGVSYNLVYSDGQGCNILKGEKGGQRGDECELWAPLGQEAHAQGSTCTAKFGEHCAAAVQHPYKADCQIPEPK
                                                    .                                .
```

Figure 8-S – Soft ticks – RGD long

```
                          10        20        30        40        50        60        70        80        90
                           |         |         |         |         |         |         |         |         |
ARGRE_58371884    MALIILLVACLSVVSADDCSGKTDAWTSIKGPKTGGYWLKQTTKTGENECTYVKGTDFKENTKTATYTYGYKDASG-----KLTKTTGTA
ORNSA_25991438    -MDCKLVAIALFIFSLDFAHAANDVWNVLKGSDSKFLMVKRTYERGANKCVYMKRTSMDESSHTLEVLMGYSKAGTTTDFVEPSKYTVTA
ARGMO_114153078   MYLLLIMLLEAATVLQADNDTRHGCDTVPRGSE--------HAEYPKTSCVYYCK-E-----EDGVWVYGYFVNDTS----FLVRHKVTA
                       ::      .         .  .  :*..          :   ..*.*    .     .      **   .         : . **

                         100       110       120       130       140       150       160       170
                           |         |         |         |         |         |         |         |
ARGRE_58371884    TAKGS------DIVVGSDTS----TVIYTDGKTCDVVKHG-----GHTELWVHSSKTSGGYNNCCDKKFTETRGSTPANEVYKKCPGMP
ORNSA_25991438    TSEGASTYNMMTVRRGPASHGVKFELVYSDDQGCNILQMKTSPFPGKCELWAPEGKAKN-VESSCSGKFKELCGDAVETPYAEGCR-VP
ARGMO_114153078   TMASA-------LSILGDIN------CPQKQKQCRRLRKPR--LDPRQDLRPPREPQLPRLKRQNKKRRKRNRRSRKRRLVSFVRHEDT
                  *  .:       :                . : *  ::        : :*           :   . : ..   .             .
```

Figure 9-S – Soft ticks - KTS

```
                          10        20        30        40        50        60        70        80        90
                           |         |         |         |         |         |         |         |         |
ARGMO_114153000   MLKLIFVAITAMVL-LDCGRGTDLF----------------------C---------------------RYPHN-----------------CQ
ARGMO_114152958   -MTPFFLALAACLVGVSHANTNDVWEAMRTSTNFYLMQRSFSKAERQCVYASINQTDSASYTYQFISGYRFPNSTWFRTAYWARAENDETGTR
                   :. :*:*::* :: :. .. .*::                      *                     *:*:.                  :

                         103       113       123       133       143       153       163       173       183
                           |         |         |         |         |         |         |         |         |
ARGMO_114153000   DSVSLSSCKG--GRVETYHYNRISRAC----IRNPLHK-----CG------------STCN-NFNS------EKQCRTSCKSYF---G----
ARGMO_114152958   TRFIVSRTRGAQGQQHDLVFSDNT-TCTILKVYNPANPSQALGCELWATLQSASSLPVVCNSTYTQNCSAIVEHNYQSTCPTVLPPPGASTT
                    . :*  :*  *: .   :.  : :*    : ** :      *              .** .:..      *:: :::* : :   *
```

Figure 10-S – Soft ticks – RTS

```
                            10        20        30        40        50        60        70
                             |         |         |         |         |         |         |
ORNCO_172051054    -MMAPTCVTFFLAFFAYLQIQEAEGFFTCVGDPSEHYYASCSKYERIKFKDRLMTCNYFCDPSKDIDCSFVEEENSPC
ARGMO_114153060    MTAAPKVVCLLCTIMC-VV---------CSGYGYDKPFGDVPKCGTVVFNWR----EIY--P----NCTYT------C
                      **. * :: :::.  :         * *   :: :.. .*   : *: *    : :  *    :*::.      *

                            88        98       108       118       128       138       148
                             |         |         |         |         |         |         |
ORNCO_172051054    WIMDYAPYGKCFKGVCYSNKTFWQATKGRESECVCDLPCEAGNDYVMTTNGDLSGGQYYCKQRPNKMCKPCGRLPDA
ARGMO_114153060    Y--ESAKVG--------DNTASWLTQKRRDDDMMC---------YVDKDRKRI-G---VCYQG---LCDDIGC----
                   :  : *  *        .*.: * : * *:.: :*         ** . .  : *    * *    :*.  *
```

Figure 11-S – Soft tick – VGD

```
                          10        20        30        40        50        60        70        80
                           |         |         |         |         |         |         |         |
ORNMO_308386965   -MLVLVTLIFSFSANIAYADSESDCTGSEPVDAFQAFSEGKEAYVLVRSTDPKARDCLKGEPAGEKQDNTLPVMMTFKNGTDWAS
ARGMO_114152986   MELKVVTALFGLAL-LSFS----------AVDS-----------LLVQDYD----GCVW-DIEGQR--------LTVGKDTFWKR
                    * :** :*.::  ::::          .**:           :**:. *    .*:  :  *::        :*. :.* *

                          95       105       115       125       135       145       155       165
                           |         |         |         |         |         |         |         |
ORNMO_308386965   TDWTFTLDGAKVTATLGNLTQNREVVYDSQSHHCHVDKVEKEVPDYEMWMLDAGGLEVEVECCRQKLEELASGRNQMYPHLKDC
ARGMO_114152986   CVYNCTLD--------GKLVLNKKMLDMSR---CQVLGTGDPLPP-TIGVCRNG------KCAKR-------------------
                    :. ***        *:*. *::::  *:   *:*  . . :*   : :   *      :*.::
```

Figure 12-S – Soft tick - MLD
